# Annonacin induces apoptosis and modulates programmed death-ligand 1 and interferon-gamma expression in triple-negative breast cancer: Integrated *in silico* and *in vitro* analysis

**DOI:** 10.14202/vetworld.2025.2241-2251

**Published:** 2025-08-09

**Authors:** Retina Yunani, Sri Agus Sudjarwo, Mustofa Helmi Effendi, Rochmah Kurnijasanti, Nurul Hidayah

**Affiliations:** 1 Doctoral Program of Veterinary Science, Faculty of Veterinary Medicine, Universitas Airlangga, Surabaya, Indonesia.; 2 Department of Clinical Pathology, Faculty of Veterinary Medicine, Universitas Wijaya Kusuma Surabaya, Surabaya, Indonesia.; 3 Department of Pharmacology, Faculty of Veterinary Medicine, Universitas Airlangga, Surabaya, Indonesia.; 4 Department of Veterinary Public Health, Faculty of Veterinary Medicine, Universitas Airlangga, Surabaya, Indonesia.; 5 Department of Microbiology, Faculty of Veterinary Medicine, Universitas Wijaya Kusuma Surabaya, Surabaya, Indonesia

**Keywords:** annonacin, apoptosis, cytotoxicity, immune modulation, *in silico*, interferon-gamma, programmed death-ligand 1, triple-negative breast cancer

## Abstract

**Background and Aim:**

Triple-negative breast cancer (TNBC) presents therapeutic challenges due to its aggressive nature and lack of targeted treatments. Programmed death-ligand 1 (PD-L1) and interferon-gamma (IFN-γ) are key immune modulators in tumor immune evasion. Annonacin, a natural acetogenin from Annona species, has shown promising anticancer properties, though its immunomodulatory mechanisms remain underexplored. This study aimed to investigate the dual apoptotic and immunomodulatory effects of annonacin on PD-L1 and IFN-γ expression using combined molecular docking and *in vitro* assays in TNBC (4T1) cells.

**Materials and Methods:**

Molecular docking simulations were conducted to assess annonacin’s interaction with PD-L1 (Protein Data Bank [PDB] ID: 6PV9) and IFN-γ (PDB ID: 1FG9). *In vitro* experiments using 4T1 cells involved 3-(4,-5- dimethylthiazol-2-yl)-2,5-diphenyltetrazolium bromide assays for cytotoxicity, Annexin V-fluorescein isothiocyanate staining for apoptosis, and flow cytometry to analyze PD-L1 and IFN-γ expression following treatment with annonacin (1.5–25 μg/mL).

**Results:**

Docking scores indicated moderate binding affinities of annonacin to IFN-γ (–5.2 kcal/mol) and PD-L1 (–5.0 kcal/mol), involving both hydrogen bonds and hydrophobic interactions. Annonacin exhibited a selective cytotoxic effect on 4T1 cells with a half-maximal inhibitory concentration of 15 μg/mL and a selectivity index of 2.6. Apoptosis was induced in a concentration-dependent manner, with late apoptotic populations peaking at 25 μg/mL. PD-L1 and IFN-γ expression peaked at 6.25 μg/mL, followed by a decline at higher doses, suggesting a dose-dependent immunomodulatory shift from immune activation to suppression.

**Conclusion:**

Annonacin modulates immune checkpoint (PD-L1) and cytokine (IFN-γ) expression while promoting apoptosis in TNBC cells. These results highlight its potential as a dual-function anticancer agent, warranting further preclinical evaluation for use as a monotherapy or in combination with immunotherapies.

## INTRODUCTION

Cancer continues to rise globally and remains a leading cause of mortality, with millions of new cases reported annually [1]. Among its hallmark features, the evasion of apoptosis and immune surveillance plays a pivotal role in cancer progression and therapeutic resistance [2]. These mechanisms not only enable tumor cells to proliferate unchecked but also compromise the efficacy of conventional treatments.

For over three decades, the induction of apoptosis has been a central strategy in clinical cancer therapy [3], with apoptosis-targeting approaches recognized as the most effective non-surgical treatments [4]. Conse-quently, the development of anticancer drugs has increasingly focused on reactivating suppressed apo-ptotic pathways or restoring their function [5], while concurrently enhancing anti-tumor immune responses through immunomodulatory strategies, such as immune checkpoint inhibition. When apoptotic mechanisms are impaired, addressing immune evasion becomes equally essential.

During cancer progression, malignant cells undergo complex adaptations to evade the body’s intrinsic tumor-suppressing mechanisms. Apoptosis inhibition is a key transformation that facilitates uncontrolled proliferation, treatment resistance, and disease recurrence [6, 7]. Both chemotherapy and immunotherapy can induce apoptosis either directly or indirectly [8]. Notably, T-cell-based immunotherapy triggers apoptosis through perforin/granzyme-mediated cytolysis, as well as ligand-induced cell death media-ted by tumor necrosis factor (TNF)-related apoptosis-inducing ligand [9].

Immunotherapy has revolutionized cancer treat-ment, surpassing traditional modalities – such as chemotherapy, radiotherapy, and surgery – in select cancers [10]. Programmed death-ligand 1 (PD-L1), a critical immune checkpoint molecule, plays an essential role in immune evasion and tumor escape, making it a prime target in cancer immunotherapy [11]. Inhibition of PD-L1 restores immune surveillance and promotes tumor eradication; notably, its downregulation enhances both spontaneous and chemotherapy-induced apoptosis in breast cancer cells [12]. PD-L1 is expressed on tumor cells, tumor-associated macrophages, and activated T lymphocytes, with its expression upregulated by pro-inflammatory cytokines, including TNFs and interferon-gamma (IFN-γ) – a key immune modulator in cancer and inflammation [13, 14]. IFN-γ has been shown to dose-dependently increase PD-L1 expression in gastric cancer cells [15], and similar associations have been documented in hepatocellular carcinoma [16].

Natural compounds continue to play a pivotal role in anticancer drug discovery [17], offering promising therapeutic alternatives with reduced toxicity and side effects. Annonacin, a bioactive acetogenin derived from *Annona* species, has demonstrated potent cytotoxicity across multiple cancer cell lines. It promotes apop-tosis through Bax upregulation, caspase-3 activation, and nuclear fragmentation, as evidenced in T24 and endometrial cancer cells [18]. However, the immu-nomodulatory potential of annonacin, particularly its effects on immune checkpoint regulation, remains lar-gely uncharacterized.

Emerging evidence suggests a close interplay between apoptotic pathways and immune signaling, particularly involving PD-L1 and IFN-γ. Therefore, this study aims to evaluate the efficacy of annonacin in inducing apoptosis while modulating PD-L1 and IFN-γ expression in triple-negative breast cancer (TNBC) cells. The study employs a combined approach of *in silico* molecular docking and *in vitro* cytotoxicity, apoptosis, and expression assays to assess the therapeutic pot-ential of annonacin. Given their ability to accelerate drug development and reduce the need for extensive preclinical screening, *in silico* approaches were utilized to predict annonacin’s molecular interactions [19], followed by experimental validation to confirm its bio-logical activity and therapeutic relevance.

Despite significant advances in immunotherapy and apoptosis-targeting treatments, TNBC remains one of the most aggressive and treatment-resistant subtypes of breast cancer, characterized by poor prognosis and limited therapeutic options. The overexpression of immune checkpoint molecules, such as PD-L1, and the dysregulation of immune cytokines, like IFN−γ, play a crucial role in immune evasion and resistance to therapy in TNBC. While monoclonal antibodies targeting programmed cell death protein 1 (PD-1)/PD-L1 have revolutionized treatment paradigms, challenges such as immune-related adverse effects, limited responsiveness, and high cost persist. Natural compounds, particularly those with dual pro-apoptotic and immunomodu-latory properties, represent a promising avenue for overcoming these limitations. Annonacin, a bioactive acetogenin derived from Annona species, has been shown to exhibit cytotoxic and pro-apoptotic effects in various cancer cell lines. However, no previous study has comprehensively explored its ability to modulate key immune checkpoint pathways, especially PD-L1 and IFN−γ, in the context of TNBC. Moreover, the mechanistic basis of its potential immunomodulatory actions – whether it activates, suppresses, or fine-tunes immune signaling – remains largely uninvestigated. This represents a critical knowledge gap, particularly considering the growing interest in plant-derived com-pounds for integrated cancer immunotherapy.

The primary aim of this study is to evaluate the dual anticancer potential of annonacin by investigating its pro-apoptotic effects and its capacity to modulate PD-L1 and IFN−γ expression in TNBC cells. Specifically, this research combines *in silico* molecular docking to predict annonacin’s binding interactions with PD-L1 and IFN−γ, with *in vitro* validation using the 4T1 mur-ine TNBC cell line to assess its cytotoxicity, apoptosis induction, and immune checkpoint modulation. By elucidating the dose-dependent effects of annonacin on apoptosis and immune signaling pathways, this study seeks to determine whether annonacin can function as an immunomodulatory agent that enhances anti-tumor responses or overcomes immune resistance in breast cancer. Ultimately, the goal is to establish a mecha-nistic foundation for further preclinical development of annonacin as a standalone or adjuvant candidate in immuno-oncology.

## MATERIALS AND METHODS

### Ethical approval

This study was approved by the Health Resea-rch Ethical Clearance Commission, Faculty of Dental Medicine, Universitas Airlangga, Indonesia (Approval No. 0056/HRECC.FODM/II/2024).

### Study period and location

The study was conducted over a 7-month period, from April to October 2024, at the Department of Parasitology and the Department of Clinical Path-ology, Faculty of Medicine, Public Health, and Nursing, Universitas Gadjah Mada (FK-KMK UGM), Yogyakarta, Indonesia.

### Study design

**Figure 1:**
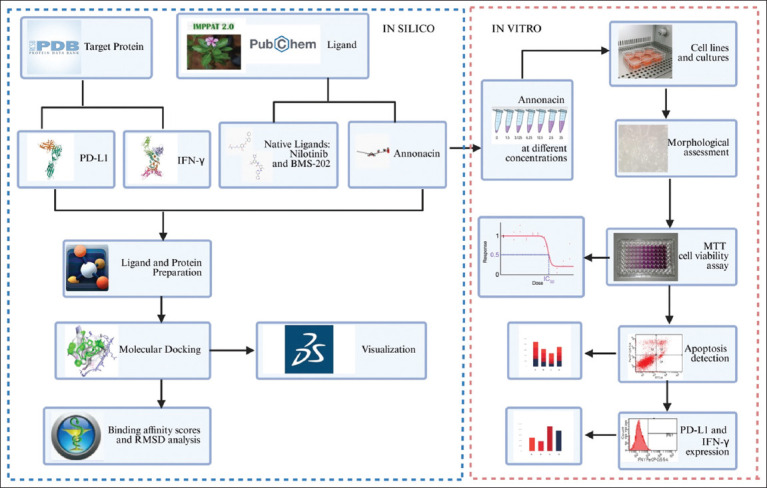
Graphical summary of the combined *in silico* screening and *in vitro* validation workflow to assess annonacin’s modulatory effects on programmed death-ligand 1 and interferon-gamma expression.

Figure 1 presents an overview of the study design and experimental workflow, integrating both *in silico* and *in vitro* analyses.

### Preparation of ligands and proteins

The molecular structure of annonacin was retrie-ved in Standard Delay Format from the Indian Medici-nal Plants, Phytochemistry and Therapeutics database (https://cb.imsc.res.in/imppat/) (ID: IMPHY003937) and converted to Protein Data Bank, Partial Charges, and Torsions format using PyRx (Scripps Research Institute, CA, USA). The crystal structures of the target proteins IFN-γ and PD-L1 (Protein Data Bank [PDB] IDs: 1FG9 and 6PV9, respectively) were obtained from the PDB (https://www.rcsb.org/). Water molecules and bound ligands were removed using PyMOL 1.7.4.5 (Schrödinger, LLC, NY, USA) Edu to expose active binding sites and avoid docking interference.

### Molecular docking procedure

Docking simulations were performed using PyRx (integrated with AutoDock Vina). The exhaustiveness level was set to 8. For each ligand, nine binding poses were generated, and the conformation with the lowest binding energy (kcal/mol) was selected for interaction analysis.

- Grid parameters:
  - *IFN-*γ *(1FG9):* Center (X = 25.7158, Y = 9.4381, Z = 13.7419); Dimensions (X = 96.7691 Å, Y = 94.7824 Å, Z = 134.7790 Å)
  - *PD-L1 (6PV9):* Center (X = −9.1350, Y = 90.9628, Z = −31.8999); Dimensions (X = 71.5928 Å, Y = 59.0200 Å, Z = 33.7371 Å).

Docked complexes were visualized using PyMOL, and binding interactions were analyzed with Disco-very Studio Visualizer 2021 (Biovia, Dassault Systèmes, CA, USA).

### Cell culture and maintenance

The 4T1 cell line (murine mammary carcinoma) was used as a TNBC model, while Vero cells (African green monkey kidney epithelial cells) served as non-cancerous controls. Both cell lines were cultured in Dulbecco’s Modified Eagle Medium supplemen-ted with 10% fetal bovine serum, 1.5% penicillin-streptomycin, and 0.5% fungizone (Sigma**-**Aldrich, Merck KGaA, Darmstadt, Germany). Cells were maintained at 37°C in a humidified incubator with 5% CO_2_. Once confluent, cells were washed with phosphate-buffered saline (PBS, pH 7.4), detached using 0.25% trypsin-ethylenediaminetetraacetic acid (EDTA), and seeded into 96-well plates at a density of 5 × 10^4^ cells/well in 100 μL medium. They were incubated for 24 h to allow monolayer formation before treatment.

### Treatment protocol and morphological assessment

Annonacin (≥95% purity; PubChem CID: 354398) was purchased from Aobious (Catalog No. AOB34570, USA). Cells were treated with various concentrati-ons of annonacin (1.5, 3.125, 6.25, 12.5, 25, 50, and 100 μg/mL) for 24 h. Untreated cells (0 μg/mL) served as negative controls. The selected dose range was based on preliminary dose-response experiments to identify effective concentrations with measurable biological responses and minimal excessive cytotoxicity. Treatments were performed in triplicate by adding 100 μL of annonacin solution to each well. Following incubation, morphological changes were examined under an inverted microscope (Carl Zeiss Axiovert 25, Germany) at 100× magnification.

### 3-(4,-5-dimethylthiazol-2-yl)-2,5-diphenyltetrazolium bromide (MTT) cell viability assay

Cell viability was measured using the MTT assay after 24 h of treatment. The culture medium was removed and replaced with 100 μL of 0.5 mg/mL MTT solution (BioVision, Inc., CA, USA), followed by incubation at 37°C for 4 h to allow formazan crystal formation. Then, 100 μL of stop solution (10% sodium dodecyl sulfate in 0.01 N HCl) was added to each well. Plates were covered with aluminum foil and left overnight at room temperature (25°C Cmp°C). Absorbance was measured at 570 nm using a Benchmark microplate reader (Bio-Rad Laboratories, Inc., CA, USA). Cell viability was calculated relative to the untreated control (0 μg/mL), which was considered 100%. All experiments were performed in triplicate. Data were processed using Microsoft Excel (version 2108; Microsoft Corporation, USA), and half-maximal inhibitory concentration (IC_50_) values were derived from dose–response curves.

### Annexin V-fluorescein isothiocyanate (FITC) apoptosis assay

Apoptosis was evaluated using Annexin V-FITC/PerCP-Cy5.5 staining and flow cytometry. 4T1 cells (5 × 10^5^ cells/well) were seeded in 6-well plates and treated for 24 h with annonacin (1.5–25 μg/mL); 0 μg/mL served as the control. After treatment, cells were harvested using 0.05% trypsin-EDTA, washed with cold PBS, and centrifuged at 300 × *g* for 5 min. Pellets were resuspended in 100 μL of 1× binding buffer, then stained with 5 μL Annexin V-FITC and 5 μL PerCP-Cy5.5 viability dye. Staining was performed in the dark at 25°C Cmp°C for 5–10 min, followed by the addition of 400 μL binding buffer. Samples were immediately analyzed using a BD FACSCanto II flow cytometer (BD Biosciences, USA). Gating strategy identified:

- Q1: Necrotic (Annexin V^-^/PerCP-Cy5.5^+^)
- Q2: Late apoptotic (Annexin V^+^/PerCP-Cy5.5^+^)
- Q3: Early apoptotic (Annexin V^+^/PerCP-Cy5.5^-^)
- Q4: Viable (Annexin V^-^/PerCP-Cy5.5^-^).

Data were analyzed using BD FACSDiva™ Software (Becton, Dickinson and Company, NJ, USA). All exper-iments were performed independently in triplicate.

### PD-L1 and IFN-γ expression assay

4T1 cells (5 × 10^5^/well) were seeded into 6-well plates and treated with annonacin (1.5–25 μg/mL) or 0 μg/mL (control) for 24 h. Cells were harvested using 0.05% trypsin-EDTA, washed with PBS, and centrifuged at 300 × *g* for 5 min. Pellets were stained using Allo-phycocyanin-A-conjugated anti-PD-L1 (E-AB-F1132E, Elabscience, USA) and FITC/PerCP-Cy5.5-conjugated anti-IFN-γ (505822, BioLegend, USA) following manu-facturer protocols. Fluorescence-minus-one and isotype controls were used to set gating thresholds. Samples were analyzed using a BD FACSCanto II flow cytometer and processed with BD FACSDiva software. Expression levels of PD-L1 and IFN-γ were expressed as percentages of positively stained cells and visualized through histo-grams and dot plots.

### Statistical analysis

Data normality was tested using the Shapiro–Wilk test, and homogeneity of variance was assessed using Levene’s test. One-way analysis of variance was performed to compare group means, followed by Tukey’s *post hoc* test for multiple comparisons when p < 0.05. Variables analyzed included proportions of viable, necrotic, early apoptotic, and late apoptotic cells, as well as PD-L1 and IFN-γ expression. All results were expressed as mean ± standard deviation with 95% confidence intervals (CI). Effect sizes were reported as partial eta squared (η^2^). All statistical analyses were conducted using IBM Statistical Package for the Social Sciences version 22 (IBM Corp., NY, USA).

## RESULTS

### *In silico* binding of annonacin to PD-L1 and IFN-γ

**Figure 2:**
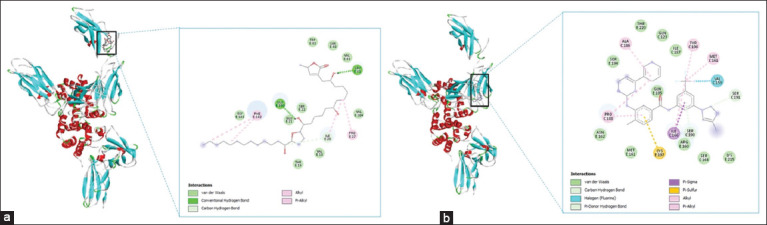
Two-dimensional and three-dimensional visualizations of molecular docking results showing (a) annonacin and (b) the native ligand (nilotinib) binding to interferon-gamma.

Molecular docking revealed that annonacin exhibited moderate binding affinity toward both IFN-γ and PD-L1, with binding energies of −5.2 kcal/mol and −5.0 kcal/mol, respectively. In the IFN-γ complex, hydrogen bonds and van der Waals forces stabilized the ligand–protein interaction. For comparison, Nilotinib – a known IFN-γ inhibitor – exhibited stronger binding through multiple non-covalent interactions, including van der Waals contacts (MET161, ASN162, SER194), carbon-hydrogen bonds, pi-donor hydrogen bonds, pi-sulfur interaction (CYS197), and halogen bonding (VAL159) (Figure 2).

**Figure 3:**
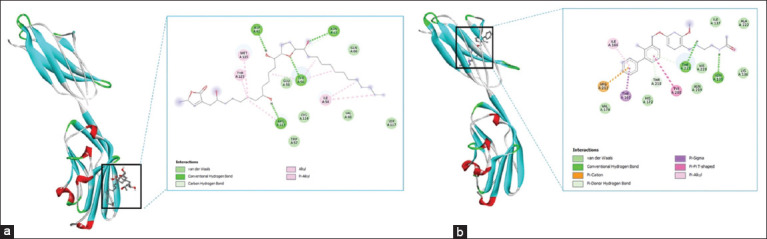
Two-dimensional and three-dimensional visualizations of molecular docking results showing (a) annonacin and (b) the native ligand Bristol-Myers Squibb compound-202 binding to programmed death-ligand 1.

Annonacin–PD-L1 binding involved four hydrogen bonds and various hydrophobic interactions, including van der Waals contacts (GLU58, GLN66, TRP57, CYS114, VAL68, and SER117), and alkyl/Pi-alkyl interactions (MET115, TYR123, and ILE54). Docking of Bristol-Myers Squibb compound 202 (BMS-202) (a reference PD-L1 inhibitor) showed strong binding through multiple con-ventional hydrogen bonds with ASN135 and THR221, a pi-donor hydrogen bond with THR210, a pi-cation interaction with ARG212, and pi–pi stacking with TYR208. Additional interactions included pi–sigma and pi–alkyl bonds with THR168 and ILE166, respectively, and van der Waals forces with residues such as ILE137, HIS172, VAL174, and HIS220 (Figure 3).

Root mean square deviation (RMSD) analysis supported the docking results. For IFN-γ (1FG9), pose 2 (ΔG = −4.9 kcal/mol, RMSD/lb = 2.52 Å) showed moderate alignment with the top-ranked pose. For PD-L1 (6PV9), pose 5 (ΔG = −4.8 kcal/mol; RMSD/lb = 2.915 Å) approached the optimal cutoff despite exceeding the ideal 2.0 Å. Nilotinib and BMS-202 exhibited higher binding affinities (−9.7 and −6.5 kcal/mol, respectively). Among Nilotinib poses, two had RMSD values below 7 Å. For BMS-202, three poses – including the second (RMSD/lb = 1.982 Å) and ninth (RMSD/lb = 2.338 Å) – were within the ideal RMSD threshold, supporting the validity of the docking predictions.

### Morphological alterations in 4T1 cells post-treatment

**Figure 4:**
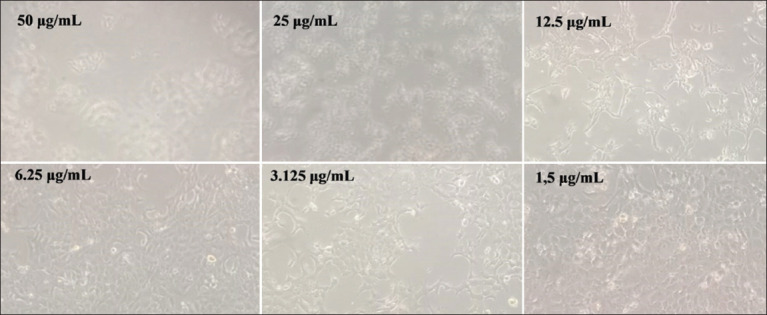
Alterations in the morphology of 4T1 cells following treatment with different concentrations of annonacin.

Following annonacin treatment, 4T1 cells exhibited dose-dependent morphological changes consistent with cytotoxicity (Figure 4). At 50 μg/mL, hallmark apoptotic features such as membrane blebbing and cell shrinkage were prominent. Intermediate concentrations (25 and 12.5 μg/mL) induced moderate cellular deformation and stress. At 6.25 and 3.125 μg/mL, minimal alte-rations were noted. The lowest dose (1.5 μg/mL) did not significantly affect cell morphology, resembling untreated controls.

### Dose-dependent inhibition of 4T1 cell proliferation

**Figure 5:**
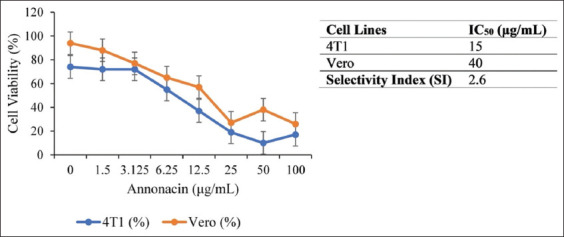
Viability of 4T1 and Vero cells after annonacin treatment, along with the half-maximal inhibitory concentration and selectivity index results.

Annonacin reduced 4T1 cell viability in a conc-entration-dependent manner (Figure 5). Viability rema-ined relatively stable at ≤3.125 μg/mL (72%–74%) but declined sharply at 6.25 μg/mL (55%) and more so at 25 μg/mL (19%) and 50 μg/mL (10%). A slight viability rebound (17%) was observed at 100 μg/mL, suggesting variability or saturation effects.

In Vero cells, annonacin caused a more gradual decline: 94% viability at baseline to 27%, 38%, and 26% at 25, 50, and 100 μg/mL, respectively. Vero cells showed greater resistance to annonacin-induced cytotoxicity. Linear regression yielded dose–response curves:

- 4T1: y = −0.5902x + 58.997 (R^2^ = 0.5661)
- Vero: y = −0.6007x + 73.743 (R^2^ = 0.6007).

The selectivity index (SI) was calculated to be 2.6, based on IC values of 15 μg/mL for 4T1 cells and 40 μg/mL for Vero cells, indicating preferential cyto-toxicity toward cancer cells.

### Annonacin-induced apoptosis in 4T1 cells

**Figure 6:**
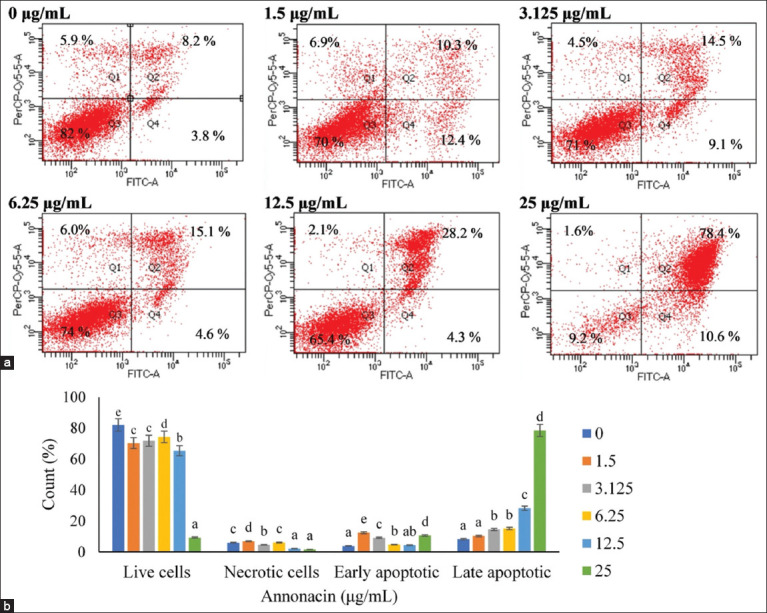
Flow cytometry analysis revealed a concentration-dependent increase in apoptotic cell populations. (a) Dot plots showing the pattern of 4T1 cells exposed to different annonacin doses. The quadrants categorize cells into four types: necrotic cells in Q1, late apoptotic cells in Q2, viable cells in Q3, and early apoptotic cells in Q4, and (b) bar graphs illustrating the percentage distribution of viable, necrotic, early apoptotic, and late apoptotic cells corresponding to the treatments.

Flow cytometry confirmed a dose-dependent increase in apoptotic cell populations following anno-nacin treatment (Figure 6). The highest viability was observed in untreated controls, while 25 μg/mL res-ulted in the lowest viability. Early apoptosis increased between 3.125 μg/mL and 6.25 μg/mL, reaching its peak in this range. Late apoptosis was most pronounced at 25 μg/mL. Across all doses, the percentages of necrotic cells remained low, further supporting apoptosis as the dominant mode of cell death. Tukey’s *post hoc* test confirmed statistically significant differences between treatment groups (p < 0.05).

### PD-L1 and IFN-γ expression response to annonacin

**Figure 7:**
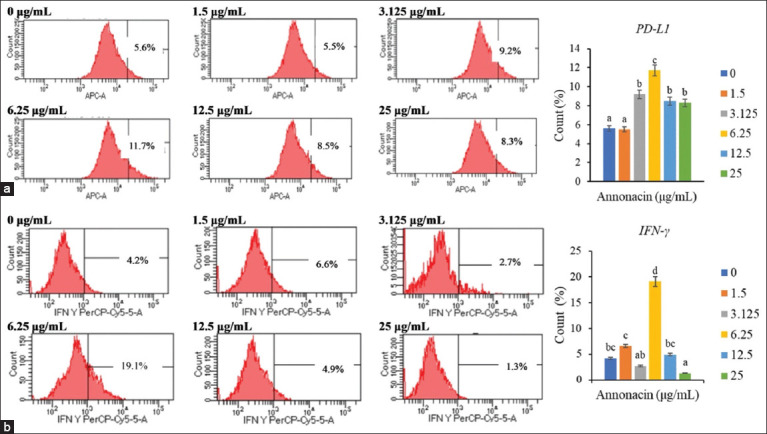
Flow cytometry analysis of protein expression following treatment with varying annonacin concentrations. Analysis focused on (a) programmed death-ligand 1 expression and (b) interferon-gamma expression.

PD-L1 and IFN-γ expression levels followed similar dose-dependent trends (Figure 7). Both markers peaked at 6.25 μg/mL and declined at higher concentrations. While PD-L1 expression decreased at 12.5 μg/mL and 25 μg/mL, it remained elevated compared to baseline. IFN-γ expression also dropped significantly at 25 μg/mL, with moderate levels observed at lower concentrations (1.5 μg/mL and 3.125 μg/mL).

Statistical analysis demonstrated a highly signifi-cant treatment effect for both PD-L1 and IFN-γ (p < 0.05).

- IFN-γ: F-test showed large effect size (partial η^2^ = 0.980). Group 6.25 had the highest mean expression (M = 19.07; 95% CI: 15.21–22.93). In contrast, expression dropped to M = 1.27 at 25 μg/mL (95% CI: 0.75–1.78).
- PD-L1: Partial η^2^ = 0.947. Peak expression was observed at 6.25 μg/mL (M = 11.73; 95% CI: 11.59–11.88), followed by a moderate decline at 12.5 μg/mL (M = 8.47; 95% CI: 6.24–10.70) and 25 μg/mL (M = 8.33; 95% CI: 6.54–10.13).

These results confirmed that annonacin modu-lates PD-L1 and IFN-γ expression in a tightly dose-dependent manner, with 6.25 μg/mL being the most immunologically responsive concentration.

## DISCUSSION

### Molecular interactions underpinning binding affinity

Binding affinity represents the strength of interaction between a ligand and its target protein, making it a crucial parameter in drug design [20]. Despite forming fewer hydrogen bonds, annonacin demonstrated stronger binding affinity toward IFN-γ (−5.2 kcal/mol) than PD-L1 (−5.0 kcal/mol), likely due to favorable hydrophobic and pi-alkyl interactions. These results highlight that factors beyond total energy, such as interaction types and geometric complementarity, have a significant influence on ligand binding.

Nilotinib, a known IFN-γ inhibitor, showed robust interaction through van der Waals forces, alkyl, pi-alkyl, and pi–sigma interactions. These non-covalent contacts, especially with aromatic residues, enhanced its stability within the binding site and lowered the binding energy. Similarly, BMS-202 exhibited strong binding to PD-L1, driven by a network of hydrogen bonds and both polar and non-polar interactions, reinforcing its role as an immune checkpoint inhibitor (ICI).

Overall, binding affinity is governed by a com-bination of van der Waals interactions, electrostat-ics, desolvation effects, and entropy [21, 22]. Docking reliability was assessed using RMSD; values exceeding 2.0 Å generally indicate reduced pose accuracy. BMS-202 showed low RMSD values, confirming stable and reliable docking poses. In contrast, IFN-γ and PD-L1 models showed higher RMSD values, indicating weaker convergence. These findings affirm the credibility of docking predictions, especially for PD-L1 interactions, and support annonacin’s potential immunomodulatory role.

### Biological relevance of predicted docking in 4T1 cells

To evaluate whether *in silico* predictions tra-nslate to biological activity, annonacin’s cytotoxic and immunomodulatory properties were assessed *in vitro* using 4T1 cells. Annonacin exhibited moderate cytotoxicity, with an IC_50_ of 15 μg/mL. Interestingly, cell viability increased slightly (17%) at 100 μg/mL, a phenomenon possibly attributable to biphasic dose-response patterns (hormesis) commonly seen in nat-ural compounds. Another plausible explanation is the aggregation or precipitation of annonacin at higher doses, which reduces cellular uptake and effective bioavailability.

This biphasic effect parallels findings in Alzheimer’s research, where Aβ (1–42) peptides require critical concentrations to aggregate and exert cytotoxic effects. Larger aggregates often fail to enter cells, thus showing reduced toxicity [23, 24]. Similarly, high dose annonacin may precipitate and fail to penetrate cells, underscoring the importance of dose optimization for therapeutic efficacy.

### Comparative cytotoxicity and selectivity toward cancer cells

Prior studies reported IC_50_ values of 21.10 μg/mL and 69.88 μg/mL for annonacin in Michigan Cancer Foundation-7 (MCF7) and T47D breast cancer cells, respectively. Coupling annonacin with nanodiamonds improved potency, lowering IC_50_ values to 22.72 μg/mL for T47D and 14.41 μg/mL for MCF7 [25]. In TNBC cell lines, annonacin inhibited proliferation, yielding IC_50_ values of 8.5 μM in MD Anderson-Metastatic Breast-468 (MDA-MB-468) and 15 μM in MDA-MB-231. Treatment also activated caspase-3, confirming induction of apoptosis [26]. In this study, annonacin’s SI of 2.6 revealed preferential cytotoxicity toward 4T1 cancer cells over Vero cells, highlighting its promise as a selectively toxic therapeutic agent, amenable to optimization through formulation or targeting strategies.

### Annonacin-induced apoptosis and morphological evidence

The pro-apoptotic effects of annonacin in 4T1 cells were validated through morphological changes consistent with apoptosis, including membrane ble-bbing, chromatin condensation, nuclear shrinkage, and pyknotic body formation. Apoptosis was supported by caspase-3 activation and DNA fragmentation [27], consistent with prior studies by Naik and Sellappan [28], who reported DNA damage in MCF-7 cells treated with annonacin.

Annexin V staining distinguished early and late apoptotic populations, showing a dose-dependent increase in late apoptosis, especially at 25 μg/mL, while necrotic cell populations remained low. Apopt-osis is a favorable pathway in cancer therapy due to its non-inflammatory nature. While necroptosis may compensate for impaired apoptosis, it often triggers inflammation, thereby complicating treatment out-comes [29]. These findings underscore annonacin’s efficacy in promoting non-inflammatory cell death at higher doses in TNBC cells.

### PD-L1 modulation and immune evasion mechanisms

Apoptosis resistance in cancer is often linked to immune escape, where PD-L1 overexpression plays a pivotal role. ICIs targeting PD-L1 have revolutionized treatment in melanoma, lung cancer, and TNBC [30, 31]. PD-L1 is highly overexpressed in TNBC and represents a major target for immunotherapeutic intervention [32].

In this study, annonacin treatment led to a dose-dependent modulation of PD-L1 expression. A slight increase was observed at 3.125 μg/mL, peaking at 6.25 μg/mL, before declining at higher concentrations. This suggests initial activation of immune evasion pathways, followed by suppression at elevated doses. Such modulation reflects findings in which polyphenols transiently upregulate PD-L1, potentially enhancing tumor visibility to ICIs [33, 34].

Meta-analyses show that combining polyphenols with ICIs can reduce PD-L1 expression more effectively than monotherapy [35]. In addition, herbs such as Astragalus membranaceus (in Buzhong Yiqi decoction) downregulate PD-L1 by suppressing phosphatidylinositol 3-kinase/Protein kinase B (PI3K/AKT) signaling in gas-tric cancer [36]. Thus, annonacin’s biphasic regulation of PD-L1 presents opportunities to use it as both an enhancer of immune recognition and a suppressor of immune resistance, depending on dose.

### Dual modulation of IFN-γ and immunostimulatory balance

IFN-γ upregulates PD-L1 expression in several cancers, promoting progression, while its receptor inhibition reduces PD-L1 levels in leukemias [37]. Although traditionally an immune-activating cytokine, IFN-γ also plays a dual role in tumors, sometimes fostering immune suppression [38].

Annonacin peaked IFN-γ expression at 6.25 μg/mL, suggesting immune activation. However, a steep decline at 25 μg/mL indicated immunosuppression, consistent with biphasic herb responses [39]. This dynamic reveals annonacin’s capacity to fine-tune immune acti-vity, promoting surveillance at moderate doses and suppressing overstimulation at high doses.

### Therapeutic relevance and translational potential

Plant-based modulators of PD-L1 and IFN-γ have shown promising effects. For instance, ginger-derived exosome-like nanoparticles increased IFN-γ and enhanced anti-PD-L1 efficacy in melanoma models [40, 41]. Similarly, triptolide downregulated IFN−γ-induced PD-L1 expression [42], and IFN−γ has been shown to mediate both apoptosis and anti-proliferative effects [43, 44].

In this study, annonacin showed concurrent peaks in PD-L1 and IFN-γ expression at 6.25 μg/mL, corresponding to enhanced viability and moderate necrosis. This reflects early immune activation followed by resistance signaling. At 25 μg/mL, increased late apoptosis and reduced necrosis were observed, coin-ciding with decreased PD-L1 and IFN-γ levels, which suggests immune exhaustion or suppression. These outcomes reinforce annonacin’s role as both an immune stimulator and suppressor, contingent on concentration.

## CONCLUSION

This study provides novel insights into the dual anticancer and immunomodulatory activities of annonacin in a TNBC model. Molecular docking sim-ulations revealed that annonacin exhibits moderate binding affinity toward PD-L1 (−5.0 kcal/mol) and IFN-γ (−5.2 kcal/mol) through multiple non-covalent interactions. These *in silico* findings were substantiated by *in vitro* assays using the 4T1 cell line, where annonacin demonstrated dose-dependent cytotoxicity, with an IC_50_ of 15 μg/mL, and a SI of 2.6, suggesting preferential toxicity toward cancer cells over normal Vero cells. Flow cytometry and morphological analyses confirmed that annonacin predominantly induces apoptosis rather than necrosis, particularly at concentrations ≥25 μg/mL. Furthermore, annonacin exhibited a biphasic effect on immune-related biomarkers. Notably, PD-L1 and IFN-γ expression peaked at 6.25 μg/mL, indicating potential activation of immune surveillance mechanisms at moderate doses. However, at higher doses (25 μg/mL), both markers declined sharply, suggesting a shift toward immunosuppression or immune exhaustion.

Annonacin’s ability to modulate both cancer cell viability and immune checkpoint pathways positions it as a promising candidate for integrative cancer therapy. Its dual role – inducing apoptosis and regulating immune signaling – could be exploited to improve outcomes in immunologically “cold” tumors like TNBC, either as a standalone agent or in combination with ICIs such as anti-PD-1 or anti-PD-L1 therapies. A major strength of this study is that it is the first to demonstrate annonacin’s modulation of PD-L1 and IFN-γ expression in TNBC. The integration of computational docking with experimental validation provides a comprehensive mechanistic framework for drug discovery. Furthermore, the use of both cancerous (4T1) and non-cancerous (Vero) cell lines strengthens the evaluation of selectivity and ther-apeutic safety.

However, this study is limited by the absence of *in vivo* validation and a lack of detailed investigation into the mechanistic pathways underlying PD-L1 and IFN-γ modulation. In addition, the biphasic responses observed at different concentrations highlight the need for further pharmacokinetic and pharmacodynamic analysis to understand dose-dependent variability and optimize therapeutic windows.

Future research should focus on validating these findings *in vivo* using relevant TNBC models and expl-oring the synergistic potential of annonacin with existing chemotherapeutics or ICIs. Investigations into its impact on key intracellular signaling pathways, such as PI3K/AKT, nuclear factor-kappa B, and janus kinase/signal transducer and activator of transcription, are also warranted. Moreover, advanced formulation strat-egies, including nanoparticle-based delivery, could help improve its bioavailability and prevent aggregation at high concentrations.

Annonacin represents a promising multi-functional bioactive compound with potential in TNBC therapy. Its dual action – cytotoxicity through apoptosis and modulation of immune checkpoints – undersco-res its translational value. With further optimization and validation, annonacin could contribute meaning-fully to the development of next-generation cancer therapies that effectively integrate cytotoxic and immunotherapeutic strategies.

## AUTHORS’ CONTRIBUTIONS

RY: Conceptualization, study design, data colle-ction, statistical analysis, manuscript drafting, and revision. SAS, MHE, and RK: Contributed to study conceptualization, design, and manuscript revision. NH: Material preparation, data collection, manuscript drafting, and revision. All authors have read and app-roved the final manuscript.
